# Effects of multimodal prehabilitation and exercise prehabilitation on patients undergoing colorectal surgery: A systematic review and meta-analysis of randomised controlled trials

**DOI:** 10.7189/jogh.14.04239

**Published:** 2024-10-25

**Authors:** Lu Zhou, Hui Li, Zhengyang Zhang, Ling Wang

**Affiliations:** 1 Department of Nursing, Peking University People’s Hospital, Beijing, China; 2 School of Nursing, Peking University, Beijing, China

## Abstract

**Background:**

Multimodal prehabilitation and exercise prehabilitation are important processes for patients undergoing colorectal surgery. There are no reviews simultaneously analysing the effects of both types of prehabilitation for patients undergoing colorectal surgery.

**Methods:**

We searched PubMed, Embase, Scopus, Web of Science, the Cochrane Library, ProQuest, and CINAHL Plus with Full Text for relevant randomised controlled trials on multimodal prehabilitation and exercise prehabilitation. The primary outcomes in our meta-analysis were functional capacity, hospital length of stay, postoperative complications, anxiety, and depression scores.

**Results:**

We included 17 studies involving 1961 for colorectal surgery patients. The results of the meta-analysis suggested that multimodal prehabilitation could improve functional capacity (the 6-minute walk test) in patients undergoing colorectal surgery (mean difference (MD) = 29.00; 95% confidence interval (CI) = 26.64–31.36). In the subgroup analysis, multimodal prehabilitation improved functional capacity only preoperatively (MD = 34.77; 95% CI = 16.76–52.77) and did not improve the length of stay, postoperative complication, and anxiety and depression scores. Exercise prehabilitation did not show a positive effect on functional capacity, the length of stay, postoperative complication, and anxiety and depression scores.

**Conclusions:**

Compared with exercise prehabilitation, multimodal prehabilitation was more likely improve the functional ability of patients undergoing colorectal surgery. Besides, the effects of multimodal prehabilitation or exercise prehabilitation on the length of stay, postoperative complications and anxiety and depression scores of colorectal surgery patients were not found.

**Registration:**

PROSPERO: CRD42023453438.

Prehabilitation is a preoperative strategy for optimising a patient's physical condition and promoting early recovery after surgery by taking relevant intervention measures before surgery [1]. It is divided into exercise, nutritional, and psychological prehabilitation [2]. The purpose of exercise prehabilitation is to enhance the exercise capacity, the physiological reserve, and functional capacity of the musculoskeletal system in patients undergoing colorectal surgery [3]. Nutritional prehabilitation, meanwhile, mainly focusses on nutritional counselling and nutrient supplementation, optimising the overall nutritional status of patients, supporting preoperative protein anabolism, and enabling patients to recover from surgery as soon as possible [4]. Lastly, psychological prehabilitation involves anxiety relief and guided relaxation training, where preoperative psychological guidance can help to reduce patients’ anxiety and depression before surgery, thereby improving their self-efficacy and improving the operative outcomes of the procedure [5,6]. Across all three subtypes, the mechanism behind the effect of prehabilitation may come from preoperative improvement of the patient's physical or psychological preparation, alteration of physiological reserves, and enhancement of functional capacity to cope with the stresses associated with surgery [7].

Prehabilitation can likewise be categorised as multimodal and unimodal [8]. As the name implies, the former consists of multiple forms of prehabilitation (e.g. exercise, nutritional, and psychological prehabilitation) [3], while the later involves only one form, such as exercise prehabilitation [9]. Multimodal prehabilitation and exercise prehabilitation are common forms of prehabilitation, but existing systematic reviews on this topic [10-12] have not covered recent studies that have found inconsistencies [13-18], nor did studies not discuss the psychological effects of prehabilitation on patients undergoing colorectal surgery. There has also been no systematic review comparing the exercise prehabilitation and multimodal prehabilitation in this population. With this gap in mind, we wanted to conduct a meta-analysis to determine the role of and conduct a comparison of exercise prehabilitation and multimodal prehabilitation in patients undergoing colorectal surgery to inform future clinical practice.

## METHODS

### Protocol registration

We conducted a systematic review based on guidelines from the Cochrane Handbook for Systematic Reviews of Interventions and reported results using the PRISMA guidelines [19]. We registered the protocol with PROSPERO (CRD42023453438).

### Data sources and search strategies

We searched PubMed, Embase, Scopus, Web of Science, the Cochrane Library, ProQuest, and CINAHL Plus with Full Text from inception to 7 July 2023 and updated the search from 1 July 2023 to 19 December 2023 after we completed the initial analysis (**Online Supplementary Document**).

We imported the retrieved records into Endnote 20 (Clarivate, London, UK) for deduplication, after which two reviewers (LZ and HL) independently screened the titles and abstracts followed by the full text of the remaining studies according to pre-determined inclusion and exclusion criteria. Any disagreements were resolved by the third author (LW).

### Inclusion and exclusion criteria

We developed our inclusion criteria according to the according to the population, intervention, comparison, outcomes, and study design (PICOS) framework, as follows:

P: Patients undergoing colorectal surgery (≥18 years old);

I: Multimodal prehabilitation or exercise prehabilitation, where all patients must have undergone at least one week of exercise prehabilitation, such as aerobic or/and resistance exercise [20];

C: Colorectal surgery patients who did not undergo multimodal prehabilitation or exercise prehabilitation before surgery;

O: The 6-minute walk test (6 MWT), postoperative complications, hospital length of stay, and anxiety and depression scores;

S: Randomised controlled trials;

We excluded) conference abstracts, study protocols, unfinished clinical trials, secondary analyses, republications, studies without full texts available, and studies missing relevant/critical data.

### Data extraction

Two researchers (LZ and HL) independently extracted the following data into a pre-developed data extraction table: publication information (author and publication year), country, study subjects, participant information (sample size, age, and population characteristics), intervention information (intervention duration, intervention measures, type of intervention, and prehabilitation site), and outcome indicators. We contacted the study authors by e-mail to get any missing data.

### Risk of bias assessment of studies and quality appraisal of evidence

We used the Cochrane Risk of Bias Tool 2.0 to assess the methodological quality of included studies independently (LZ and ZZ) [21]. The Grading of Recommendations, Assessment, Development and Evaluation framework classifies the certainty of evidence is classified as high, moderate, low, or very low [22], where the quality is downgraded based on five factors: study limitations (risk of bias), inconsistent findings, indirect evidence, imprecise findings, and biased reporting [23]. In case of disagreement, a third appraiser was included (LW).

### Data analysis

We performed the meta-analysis using RevMan, version 5.4 (Cochrane, London, UK). We presented the effect sizes of dichotomous outcomes (e.g. postoperative complication) as risk ratio (RRs) and continuous outcomes (e.g. 6MWT, anxiety and depression scores, or length of stay) as mean differences (MDs) or standardised mean difference (SMDs), with corresponding 95% confidence intervals (CIs) for all variables. We used Cochran’s Q to assess statistical heterogeneity, with the *I*^2^ representing inter-study heterogeneity, where by *I*^2^ values of 25%, 50% and 75%, indicated low, medium and high heterogeneity, respectively [21]. We therefore used random effects models if the *P* < 0.1 or *I*^2^ > 50% and fixed effects models if *P* ≥ 0.1 and *I*^2^ ≤ 50% [24]. For studies with non-parametric results, we calculated mean and standard deviation (SD) by dividing the interquartile range (IQR) by 1.35 [21].

We performed analyses by type of intervention (multimodal or exercise prehabilitation), subgroup analyses for 6MWT according to outcome measurement time, and sensitivity analyses according to population characteristics and risk bias of included studies. When the meta-analysis contained more than ten studies, we analysed publication bias using funnel plots [21].

## RESULTS

### Search results

**Figure 1:**
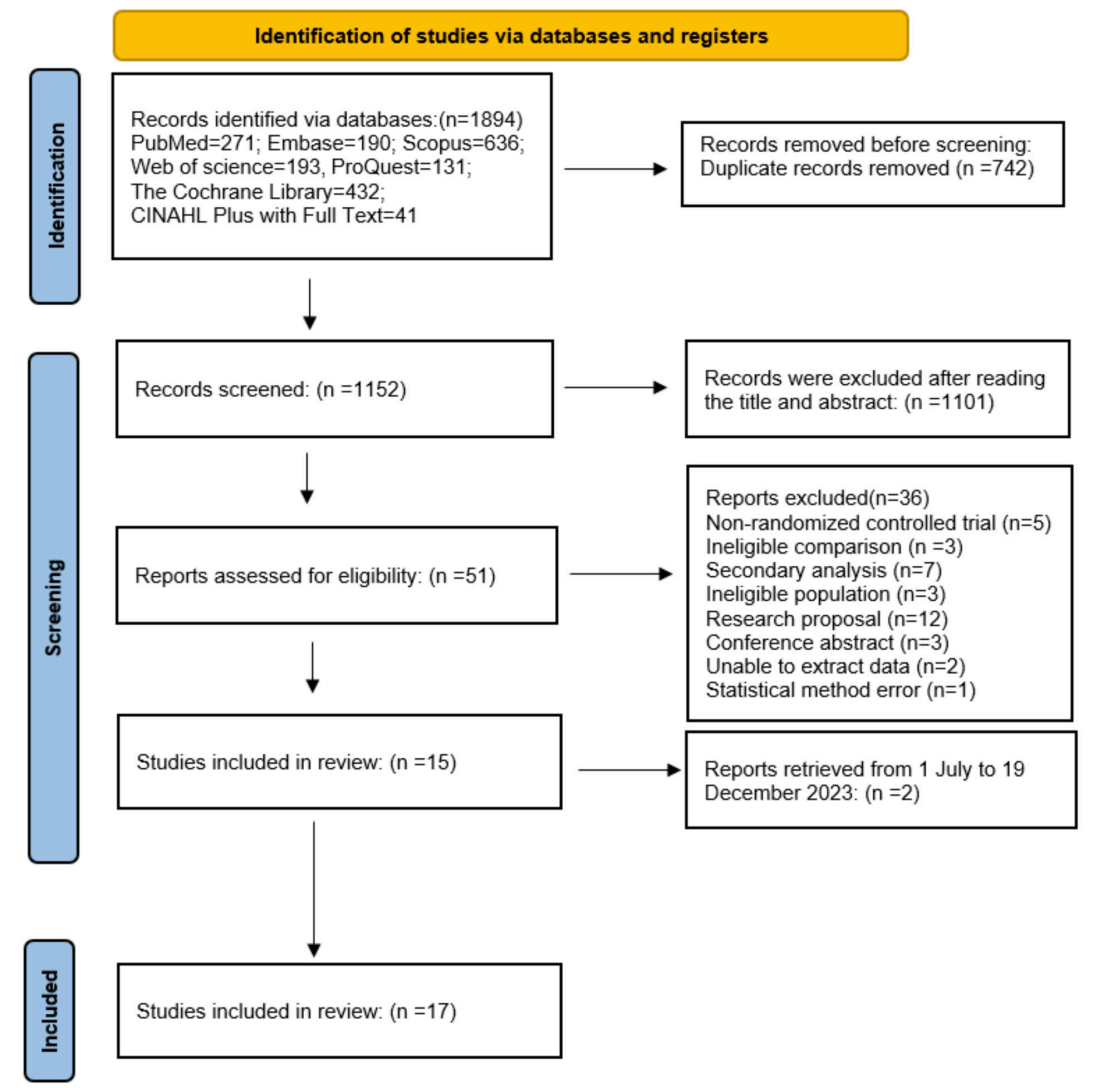
Study flowchart, adapted from the PRISMA guidelines [19].

Our search retrieved 1894 studies, of which we excluded 742 as duplicates and 1101 after the title and abstract screening (**Figure 1**). We excluded a further 36 studies at the full-text screening stage, leaving 15 studies in our analysis [9,13-18,25-32]. We updated the search time from July 1 to December 19, 2023, retrieving a total of 101 records, from which we excluded 21 duplicates, 70 ineligible studies following title and abstract screening, and an additional eight during full-text screening, leaving two studies for inclusion [33,34]. This resulted in 17 studies being included in the analysis.

### Study characteristics

In these 17 studies, 965 patients received and 996 patients did not receive a prehabilitation intervention. These interventions varied in duration (range: 2–14 weeks) and in sample size (range: 20–674) (**Table 1**).

**Table 1:** Study characteristics

Author, year (reference)	Country	Population characteristics	Number of patients in IC/CG	Age of patients in IC/CG	Intervention measure – IG	Intervention measure - CG	Duration of prehabilitation	Prehabilitation type	Prehabilitation site	Outcome
Carli et al., 2020 [13]	Canada	Frail patients undergoing resection of colorectal cancer	55/55	MD = 78 (IQR = 72–82)/MD = 82 (IQR = 75–84)	Exercise intervention (aerobic exercise, resistance exercise), nutrition intervention, psychological intervention	Interventions were consistent with the prehabilitation group, but started after surgery	Four weeks	Multimodal	Hospital and home	LOS, complications, 6MWT, HADS
Molenaar et al., 2023 [14]	The Netherlands	Adult patients scheduled for elective surgical resection of primary colorectal cancer	123/128	MD = 69 (IQR = 60–77)/MD = 71 (IQR = 60–76)	Exercise intervention (aerobic and strength exercises), nutrition intervention, psychological intervention	Enhanced recovery after surgery	Four weeks	Multimodal	Hospitals and care centers	LOS, complications, 6MWT
Fulop et al., 2021 [25]	Hungary	Adult patients who required colorectal resection surgery	77/72	MD = 70 (IQR = 60–75)/MD = 70 (IQR = 64–75)	Exercise intervention (aerobic and breathing exercises), nutrition intervention, psychological intervention	Enhanced recovery after surgery	Three to six weeks	Multimodal	Hospital and home	LOS, 6MWT, HADS
Berkel et al., 2022 [15]	The Netherlands	High-risk patients scheduled for elective colorectal resection for (pre)malignancy	28/29	x̄ = 74 (SD = 7)/x̄ = 73 (SD = 6)	Exercise programme (aerobic exercise, resistance exercise)	Usual care	Three weeks	Exercise	Community	LOS, complication
Gillis et al., 2014 [26]	Canada.	Adult patients scheduled for curative resection of nonmetastatic colorectal cancer	38/39	x̄ = 65.7 (SD = 13.6)/x̄ = 66.0 (SD = 9.1)	Exercise intervention (aerobic and resistance training), nutrition intervention, psychological intervention	Interventions were consistent with the prehabilitation group, but started after surgery	MD = 24.5 d	Multimodal	A single university affiliated tertiary center	LOS, complications, 6MWT, HADS
Peng et al., 2021 [16]	China	Patients scheduled for elective colorectal surgeries	109/104	x̄ = 63.0 (SD = 2.8)/x̄ = 62.8 (SD = 3.1)	Strengthening of the upper and lower extremities; thoracic and abdominal breathing exercises; exercise of abdominal muscles	Enhanced recovery after surgery	x̄ = 14.3 d (SD = 5.2)	Exercise	Hospital and home	LOS
Moug et al., 2019 [9]	UK	Patients with rectal cancer undergoing neoadjuvant chemoradiotherapy and scheduled for colon or rectal cancer resection	18/22	x̄ = 65.2 (SD = 11.4)/x̄ = 66.5 (SD = 9.6)	Physical activity(walking)	Usual care	MD = 14 weeks (IQR = 13–17)	Exercise	Home	6MWT, complications, BDI-II
Bousquet-Dion et al., 2018 [27]	Canada	Adult patients scheduled for colon or rectal cancer resection	37/26	MD = 74 (IQR = 67.5–78)/MD = 71 (IQR = 54.5–74.5)	Exercise intervention (aerobic and resistance training), nutrition intervention, psychological intervention	Interventions were consistent with the prehabilitation group, but started after surgery	Four weeks	Multimodal	Hospital and home	LOS, complications, 6MWT
López-Rodríguez-Arias et al., 2021 [28]	Spain	Patients undergoing elective surgery for colon or rectal neoplasm	10/10	MD = 66 (IQR = 61.8–71.5)/MD = 66.5 (IQR = 57.7–70)	Exercise intervention (aerobic and muscular resistance training), nutrition intervention, psychological intervention	Usual care	x̄ = 28.9 d (SD = 2.8)	Multimodal	Home	LOS, complications, HADS
Northgraves et al., 2019 [17]	UK	Patients were listed for major elective colorectal cancer resection surgery	10/11	x̄ = 64.1 (SD = 10.5)/x̄ = 63.5 (SD = 12.5)	Both aerobic and resistance training	Usual care	x̄ = 22.0 d (SD = 7.5)	Exercise	Hospital	LOS, 6MWT
Taha et al., 2021 [18]	Switzerland	Patients with colorectal cancer	23/25	x̄ = 64.8 (SD = 11.5)/x̄ = 64.0 SD = 11.9)	Both aerobic and resistance training	Usual care	Three to six weeks	Exercise	Institute of physiotherapy and home	HADS
Chen et al., 2016 [30]	Canada	Elderly patients scheduled for colorectal cancer surgery	57/59	x̄ = 67.9 (SD = 1.5)/x̄ = 67.3 (SD = 1.2)	Including exercise intervention (aerobic and resistance training), nutrition intervention, psychological intervention	Interventions were consistent with the prehabilitation group, but started after surgery	Four weeks	Multimodal	Hospital	6MWT
Kim et al., 2009 [29]	Canada	Patients undergoing bowel resection surgery	14/7	x̄ = 55 (SD = 15)/x̄ = 65 (SD = 9)	Aerobic exercise	Usual care	Four weeks	Exercise	Hospital	6MWT
Onerup et al., 2022 [31]	Sweden	Patients were listed for major elective colorectal cancer resection surgery	317/357	x̄ = 69 (SD = 11)/x̄ = 68 (SD = 11)	Aerobic exercise	Usual care	Two weeks	Exercise	Hospital and home	LOS, complications
Karlsson et al., 2019 [32]	Sweden	Scheduled for surgery due to colorectal cancer or suspected colorectal cancer	10/11	MD = 83.5 (IQR = 76–85)/MD = 74 (IQR = 73–76)	Inspiratory muscle training, functional strength exercises, endurance training	Usual care and advice to follow the recommendation of 150 min/week of moderate physical activity	More than two weeks	Exercise	Hospital	LOS, complications
Triguero-Cánovas et al., 2023 [33]	Spain	Patients treated surgically for colorectal cancer.	23/21	x̄ = 68.09 (SD = 7.67)/x̄ = 67.24 (SD = 8.51)	Physical activity, nutritional supplementation, and relaxation exercises	Usual care	Six to eight weeks	Multimodal	Home	6MWT, LOS, complications
Bojesen et al., 2023 [34]	Denmark	Patients with colorectal cancer surgery	16/20	x̄ = 80 (SD = 6.9)/x̄ = 78 (SD = 6.3)	Physical activity, nutritional supplementation, medical optimisation prior to surgery	Usual care	MD = 32 d (IQR = 29–34 d) between inclusion and surgery for the IG and MD = 24 d (IQR = 18–28 d) for the CG	Multimodal	Hospital	6MWT, LOS, complications

6MWT – 6-Minute Walk Test, BDI-II-Beck Depression Index-II depression scale, CG – control group, HADS – Hospital Anxiety and Depression Scale, IG – intervention group, IQR – interquartile range, LOS – length of stay, SD – standard deviation, x̄ – mean

### Risk of bias assessment

Eight studies [9,13-18,26] had a low risk, while nine had ‘some concerns’ due to not reporting their allocation sequence (all nine studies) [25,27-34] or having no preregistration (one study) [29] (Table S1 in the **Online Supplementary Document**).

### Results of syntheses

#### Effect of prehabilitation on functional capacity

Eleven studies (n = 886) explored the effects of exercise prehabilitation and multimodal prehabilitation on the 6MWT in patients undergoing colorectal surgery (*I*^2^ = 14%, *P* = 0.310), with the fixed effects model indicating effective improvement of the interventions (MD = 29.00; 95% CI = 26.64, 31.36) (Figures S1 in the **Online Supplementary Document**). However, the subgroup analysis conversely found that exercise prehabilitation could not effectively improve the 6MWT of colorectal surgery patients (MD = 13.24; 95% CI = −37.89, 64.36), while the findings for multimodal prehabilitation remained stable (MD = 29.03; 95% CI = 26.67, 31.39). Based on the measurement time, we further analysed the impact of multimodal prehabilitation on the 6MWT of colorectal surgery patients before, four weeks after surgery, and six to eight weeks after surgery, and found that their 6MWT improved before surgery (MD = 34.77; 95% CI = 16.76, 52.7), but did not improve four weeks (MD = 10.17; 95% CI = –9.82, 30.16) and six to eight weeks after surgery (MD = 17.78; 95% CI = −20.77, 56.33) (Figures S2 and S3 in the **Online Supplementary Document**).

#### Effect of prehabilitation on hospital length of stay

Based on 13 studies (n = 1730), the fixed effects model (*I*^2^ = 0%, *P* = 0.570) showed that both methods do not effectively reduce the length of hospital stay in colorectal surgery patients (MD = −0.14; 95% CI = −0.31, 0.03) (Figure S4 in the **Online Supplementary Document**). These findings were confirmed in the subgroup analysis (MD = −0.11, 95% CI = −0.34, 0.12 for multimodal prehabilitation, MD = −0.18; 95% CI = −0.42, 0.07 for exercise prehabilitation) (Figure S5 in the **Online Supplementary Document**).

#### Effect of prehabilitation on postoperative complications

Eleven studies (n = 1387) analysed the effects of exercise prehabilitation and multimodal prehabilitation on complications in patients undergoing colorectal surgery. The random effects model (*I*^2^ = 56%, *P* = 0.010) showed no reduction due to either intervention (RR = 0.87; 95% CI = 0.71, 1.07) (Figure S6 in the **Online Supplementary Document**). The subgroup analysis, in turn, confirmed the findings for exercise prehabilitation (RR = 1.03; 95% CI = 0.70, 1.51), but determined that multimodal prehabilitation (RR = 0.78; 95% CI = 0.65, 0.94) can effectively reduce the complications of colorectal surgery patients (Figure S7 in the **Online Supplementary Document**).

#### Effect of prehabilitation on anxiety and depression scores

Five studies (n = 382) analysed the effects of exercise prehabilitation and multimodal prehabilitation on anxiety in patients undergoing colorectal surgery, all using the Hospital Anxiety and Depression Scale (HADS). The fixed effects model (*I*^2^ = 0%, *P* = 0.890) found that both exercise prehabilitation and multimodal prehabilitation can relieve anxiety in this group (MD = −0.71; 95% CI = −1.41, −0.01) (Figure S8 in the **Online Supplementary Document**). The findings were confirmed by the subgroup analysis for multimodal prehabilitation (MD = −0.76; 95% CI = −1.53, 0.00), but not the one for exercise prehabilitation (MD = −0.40; 95% CI = −2.21, 1.41) (Figure S9 in the **Online Supplementary Document**).

Of the six studies (n = 419) exploring the effects of exercise prehabilitation and multimodal prehabilitation on depression in patients undergoing colorectal surgery, five used the HADS and one used the Beck Depression Index-II depression scale. The fixed effects model (*I*^2^ = 0%, *P* = 0.290) showed no effect on the interventions in terms of depression score reduction (SMD = −0.10; 95% CI = −0.30, 0.09) (Figure S10 in the **Online Supplementary Document**), which was further confirmed in the subgroup analysis (SMD = 0.04; 95% CI = −0.39, 0.47 for exercise prehabilitation, (SMD = −0.14; 95 % CI = −0.36, 0.07 for multimodal prehabilitation) (Figure S11 in the **Online Supplementary Document**).

### Sensitivity analysis and assessment of publication bias

Our findings remained stable in our sensitivity analysis by population characteristics, we excluded studies in high-risk populations (such as frail patients, elderly patients, or patients with neoadjuvant chemoradiotherapy). In our sensitivity analysis by risk bias of the studies, we excluded studies that had ‘some concerns’; in contrast to our initial findings, we observed no effect of multimodal prehabilitation reduces anxiety scores and postoperative complications in patients undergoing colorectal surgery. The other combined results of the meta-analysis did not change significantly in this sensitivity analysis. Visually, the funnel plots were evenly distributed and symmetrical, indicating a low probability of publication bias (Table S2 and Figure S12 in the **Online Supplementary Document**).

### Certainty of the evidence

We found moderate-quality evidence for the impact of the interventions on hospital length of stay (exercise and multimodal prehabilitation) and complications (multimodal prehabilitation only); low-quality evidence for 6MWT (multimodal prehabilitation only), complications (exercise prehabilitation only), anxiety scores (exercise and multimodal prehabilitation), depression scores (exercise and multimodal prehabilitation), and very low-quality evidence for 6MWT (exercise prehabilitation) (Table S3 in the **Online Supplementary Document**).

## DISCUSSION

### Functional capacity

We found that, in contrast to exercise prehabilitation, multimodal prehabilitation could effectively improve patients' 6MWT before surgery. There may be several underlying mechanisms. First, we found that exercise-based prehabilitation for undergoing colorectal surgery patients is relatively simple, as it is mainly based on aerobic exercise. Previous studies have found that resistance exercise helps promote muscle production and enhance patients’ endurance [35,36], and that aerobic exercise combined with resistance training may be better for improving patients’ functional capacity [37]. Moreover, our study team previously carried out a dietary, multimodal prehabilitation-based intervention which can effectively improve the protein intake of patients undergoing colorectal surgery, stimulate muscle synthesis, and ultimately improve their functional capacity [38,39]. Similarly, a recently published systematic review [40] found that multimodal prehabilitation improved functional ability in this population. Based on these findings, we generally we recommend multimodal rather than exercise prehablitation for colorectal surgery patients.

We also analysed the effects of multimodal prehabilitation intervention on 6MWT in colorectal surgery patients at different time points and found that it could improve 6MWT before surgery, but not post-surgery. This is, to an extent, in line with prior studies. For example, Molenaar et al. [10] found that preoperative multimodal prehabilitation can improve patients’ functional capacity of patients. However, in our meta-analysis, the effect of multimodal prehabilitation on 6MWT of colorectal surgery patients was not significant. This may be related to the enhanced recovery after surgery, which can seemingly help improve the functional capacity of colorectal surgery patients and reduce the occurrence of complications [41,42], thereby masking the role of multimodal prehabilitation in this context.

### Postoperative complications

We found that multimodal prehabilitation and exercise prehabilitation had limited effect on reducing postoperative complications in patients. This may be related to multiple reasons. First, postoperative intervention, such as enhanced recovery after surgery, can reduce the occurrence of postoperative complications [43], limiting the effect of exercise or multimodal prehabilitation in this sense. We observed that the time of preoperative prehabilitation was not very long, and that the reduction of complications in colorectal surgery patients was difficult to achieve through short-term prehabilitation [13]. Most of the included studies, however, only reported the number of postoperative complications and did not indicate their severity, so we only performed meta-analysis on the number of complications. However, in their study of 251 colorectal surgery patients, Molenaar et al. [14] found that multimodal prehabilitation did not improve the number of postoperative complications in colorectal cancer patients. This is consistent with our findings; however, in further subanalyses, the aforementioned study found that multimodal prehabilitation had a positive significance in reducing the severity of complications [14]. Future research should further group patients according to the severity of their complications to further clarify the effectiveness of different types of prehabilitation in reducing postoperative complications.

### Length of hospital stay

We found that multimodal and exercise prehabilitation had limited effect in terms of improving hospital stay in patients undergoing colorectal surgery. The length of hospital stay is closely related to the occurrence of complications; here we determined that both types of prehabilitation could not effectively reduce the occurrence of complications. Moreover, the continuous development of laparoscopic technology resulted in laparoscopic surgery leading to less trauma for colorectal patients and shorter postoperative recovery time, which further helps to reduce the length of hospital stay [44]. Yet in contrast, the meta-analysis of colorectal surgery patients by Marmol et al. [40] showed that prehabilitation helped reduce the length of hospitalisation. This inconsistency with our findings might be due to the different designs of the included studies, as we exclusively included randomised controlled trials. Given these discrepant findings, we cannot disregard the role of prehabilitation in reducing the length of hospital stay; further research is warranted to clarify this relationship.

### Anxiety and depression scores

We found no effect of exercise prehabilitation and multimodal prehabilitation on anxiety and depression scores in colorectal surgery patients. This is the first meta-analysis to explore this relationship. Existing studies have previously had inconsistent findings. For example, in a study of 149 colorectal surgery patients, Lim et al. [25] found that multimodal prehabilitation can help reduce the anxiety level of patients. There might be several mechanism at play here. First, the baseline anxiety and depression scores of the colorectal surgery patients in the included studies were mostly at normal levels, and it was difficult to further reduce the anxiety and depression scores of patients through prehabilitation. Finally, there are few studies analysing the effects of prehabilitation on anxiety and depression in this population in general, so we could not find strong evidence to explore this in our meta-analysis. Future primary studies will need to further clarify these effects.

### Strengths, limitations, and recommendations

The meta-analysis was the first to integrate and compare the effects of exercise prehabilitation and multimodal prehabilitation on patients undergoing colorectal surgery: The included in this meta-analysis were all randomised controlled trials; none were at high risk of bias and all had low likelihood of publication bias. Moreover, we explored and discussed the psychological and other effects of exercise rehabilitation and multimodal rehabilitation in colorectal surgery patients, which could inform the work of clinicians with this population.

However, we have to highlight some limitations. Due to the shortcomings of the included studies, we could compare the effects of multimodal prehabilitation and exercise prehabilitation on patient outcomes only indirectly. Future studies should apply eligibility criteria more evenly to ensure the inclusion of comparable groups of patients, allowing for more robust interpretability and useability of their results. Lastly, only a small number of studies identified the preoperative physiological status of patients; future studies should make such distinctions, as these difference may affect the effect of prehabilitation.

## CONCLUSIONS

This systematic review and meta-analysis show that multimodal prehabilitation can improve the functional capacity of patients before surgery, but not post-surgery. We also observed no role of exercise prehabilitation in improving the functional ability of patients undergoing colorectal. The effects of both types of prehabilitation on the length of hospital stay, postoperative complications, and anxiety and depression scores were not significant. Future research should focus on large-scale, high-quality randomised controlled trials to further clarify the impact of these interventions on patients undergoing colorectal surgery.

## Additional material


Online Supplementary Document
