# Supplementary material for: Effects of multimodal prehabilitation and exercise prehabilitation on patients undergoing colorectal surgery: A systematic review and meta-analysis of randomised controlled trials
Source: J Glob Health. 2024 Oct 25;14:04239. doi: 10.7189/jogh.14.04239 (PMC11505574; doi:10.7189/jogh.14.04239)
Supplement: Online Supplementary Document [file jogh-14-04239-s001.pdf]

Specific search strategies

PubMed

#1：(((((Colorectal[Title/Abstract]) OR (colonic[Title/Abstract])) OR (colon[Title/Abstract])) OR (rectal[Title/Abstract])) OR (rectum[Title/Abstract])) OR (sigmoid[Title/Abstract])

#2：((((((exercise[Mesh]) OR (exercise[Title/Abstract])) OR (physical[Title/Abstract])) OR (training[Title/Abstract])) OR (nutri*[Title/Abstract])) OR (Psych*[Title/Abstract])) OR (mental[Title/Abstract])

#3：(((((((prior to surgery[Title/Abstract]) OR (prior to operation[Title/Abstract])) OR (preoperative[Title/Abstract])) OR (pre-operation[Title/Abstract])) OR (prehabilitation[Title/Abstract])) OR (pre-habilitation[Title/Abstract])) OR (presurgical[Title/Abstract])) OR (pre-surgical[Title/Abstract])

#4：(((((Clinical Trial [Publication Type]) OR (Randomized Controlled Trial[Publication Type])) OR (random allocation[Mesh])) OR (randomized controlled trial*[Title/Abstract])) OR (random* allocation[Title/Abstract])) OR (clinical trial*[Title/Abstract])

#5：#1 AND #2 AND #3 AND #4

Embase

#1: colorectal:ti,ab,kw OR colonic:ti,ab,kw OR colon:ti,ab,kw OR rectal:ti,ab,kw OR rectum:ti,ab,kw OR sigmoid:ti,ab,kw

#2: 'prior to surgery':ti,ab,kw OR 'prior to operation':ti,ab,kw OR preoperative:ti,ab,kw OR 'pre operation':ti,ab,kw OR prehabilitation:ti,ab,kw OR 'pre habilitation':ti,ab,kw OR presurgical:ti,ab,kw OR 'pre surgical':ti,ab,kw

#3：exercise:ti,ab,kw OR physical:ti,ab,kw OR training:ti,ab,kw OR nutri*:ti,ab,kw OR psych*:ti,ab,kw OR mental:ti,ab,kw

#4: 'randomized controlled trial*':ti,ab,kw OR 'random* allocation':ti,ab,kw OR 'clinical trial*':ti,ab,kw

#5:#1 AND #2 AND #3 AND #4

Scopus

#1：(TITLE-ABS-KEY (colorectal) OR TITLE-ABS-KEY (colonic) OR TITLE-ABS-KEY (colon) OR TITLE-ABS-KEY (rectum) OR TITLE-ABS-KEY (sigmoid)) 744596

#2：(TITLE-ABS-KEY (exercise) OR TITLE-ABS-KEY (physical) OR TITLE-ABS-KEY (training) OR TITLE-ABS-KEY (nutri*) OR TITLE-ABS-KEY (psych*) OR TITLE-ABS-KEY (mental))

#3：(TITLE-ABS-KEY ("prior to surgery") OR TITLE-ABS-KEY ("prior to operation") OR TITLE-ABS-KEY (preoperative) OR TITLE-ABS-KEY (pre-operation) OR TITLE-ABS-KEY (prehabilitation) OR TITLE-ABS-KEY (pre-habilitation) OR TITLE-ABS-KEY (presurgical) OR TITLE-ABS-KEY (pre-surgical))

#4：(TITLE-ABS-KEY ("randomized controlled trial* ") OR TITLE-ABS-KEY ("random* allocation") OR TITLE-ABS-KEY ("clinical trial*"))

#5：#1 AND #2 AND #3 AND #4

Web of science

#1：(((((AB=(Colorectal)) OR AB=(colonic)) OR AB=(colon)) OR AB=(rectal)) OR AB=(rectum)) OR AB=(sigmoid)

#2：(((((AB=(exercise)) OR AB=(physical)) OR AB=(training)) OR AB=(nutri*)) OR AB=(Psych*)) OR AB=(mental)

#3：(((((((AB=(prior to surgery)) OR AB=(prior to operation)) OR AB=(preoperative)) OR AB=(pre-operation)) OR AB=(prehabilitation)) OR AB=(pre-habilitation)) OR AB=(presurgical)) OR AB=(pre-surgical)

#4：((AB=(randomized controlled trial*)) OR AB=(random* allocation)) OR AB=(clinical trial*)

#5： #1 AND #2 AND #3 AND #4

The Cochrane Library

#1 (Colorectal):ti,ab,kw OR (colonic):ti,ab,kw OR (colon):ti,ab,kw (Word variations have been searched)

#2 (rectum):ti,ab,kw OR (sigmoid):ti,ab,kw (Word variations have been searched)

#3 #1 OR #2

#4 MeSH descriptor: [Exercise] explode all trees

#5 ("prior to surgery"):ti,ab,kw OR ("prior to operation"):ti,ab,kw OR (preoperative):ti,ab,kw OR (pre-operation):ti,ab,kw (Word variations have been searched)

#6 (prehabilitation):ti,ab,kw OR (pre-habilitation):ti,ab,kw OR (presurgical):ti,ab,kw OR (pre-surgical):ti,ab,kw (Word variations have been searched)

#7 #5 OR #6

#8 (exercise):ti,ab,kw OR (physical):ti,ab,kw OR (training):ti,ab,kw (Word variations have been searched)

#9 (nutri*):ti,ab,kw OR (Psych*):ti,ab,kw OR (mental):ti,ab,kw (Word variations have been searched)

#10 #4 OR #8 OR #9

#11 MeSH descriptor: [Randomized Controlled Trial] explode all trees

#12 MeSH descriptor: [Random Allocation] explode all trees

#13 MeSH descriptor: [Clinical Trial] explode all trees

#14 ("randomized controlled trial"):ti,ab,kw OR ("random allocation"):ti,ab,kw OR ("clinical trial"):ti,ab,kw (Word variations have been searched)

#15 #11 OR #12 OR #13 OR #14

#16 #3 AND #7 AND #10 AND #15

ProQuest

#1: abstract(Colorectal) OR abstract(colonic) OR abstract(colon) OR abstract(rectal) OR abstract(rectum) OR abstract(sigmoid)

#2: abstract("prior to surgery") OR abstract("prior to operation") OR abstract(preoperative) OR abstract(pre-operation) OR abstract(prehabilitation) OR abstract(pre-habilitation) OR abstract(presurgical) OR abstract(pre-surgical)

#3: abstract(exercise) OR abstract(physical) OR abstract(training) OR abstract(nutri*) OR abstract(Psych*) OR abstract(mental)

#4: abstract("randomized controlled trial* ") OR abstract("random* allocation") OR abstract("clinical trial*")
#5: #1 AND #2 AND #3 AND #4

CINAHL Plus with Full Text
#1: AB Colorectal OR AB colonic OR AB colon OR AB rectal OR AB rectum OR AB sigmoid
#2: AB "prior to surgery " OR AB "prior to operation" OR AB preoperative OR AB pre-operation OR AB prehabilitation OR AB pre-habilitation OR AB presurgical OR AB pre-surgical
#3: AB exercise OR AB physical OR AB training OR AB nutri* OR AB Psych* OR AB mental
#4: AB "randomized controlled trial* " OR AB "random* allocation" OR AB " clinical trial*"
#5: #1 AND #2 AND #3 AND #4

Figure S1: Overall meta-analysis-6MWT

Study or Subgroup	Experimental Mean	Experimental SD	Experimental Total	Control Mean	Control SD	Control Total	Weight	Mean Difference IV, Fixed, 95% CI
Bojesen et al 2023	304	110.37	16	260	75.59	20	0.1%	44.00 [-19.42, 107.42]
Bousquet-Dion et al 2018	468	118	37	472	108	26	0.2%	-4.00 [-60.29, 52.29]
Carli et al 2020	336.4	121.8	38	286.1	105.1	30	0.2%	50.30 [-3.68, 104.28]
Chen et al 2016	23.7	6.9	57	-5.4	6.2	59	97.5%	29.10 [26.71, 31.49]
Fulop et al 2021	278	143	77	300	151.1	72	0.2%	-22.00 [-69.31, 25.31]
Gillis et al 2014	23.4	54.8	38	-21.8	80.7	39	0.6%	45.20 [14.46, 75.94]
Kim et al 2009	467	80	14	504	103	7	0.1%	-37.00 [-124.05, 50.05]
Molenaar et al 2023	536	98.5	123	514	103.7	128	0.9%	22.00 [-3.01, 47.01]
Moug et al 2019	462.5	144.3	18	390.1	159.4	22	0.1%	72.40 [-21.84, 166.64]
Northgraves et al 2019	473.7	93	10	460.7	106	11	0.1%	13.00 [-72.13, 98.13]
Triguero-Cánovas et al 2023	372.1	115.7	23	313.9	154.2	21	0.1%	58.20 [-22.95, 139.35]
**Total (95% CI)**			**451**			**435**	**100.0%**	**29.00 [26.64, 31.36]**

Heterogeneity: Chi² = 11.63, df = 10 (P = 0.31); I² = 14%
Test for overall effect: Z = 24.09 (P < 0.00001)

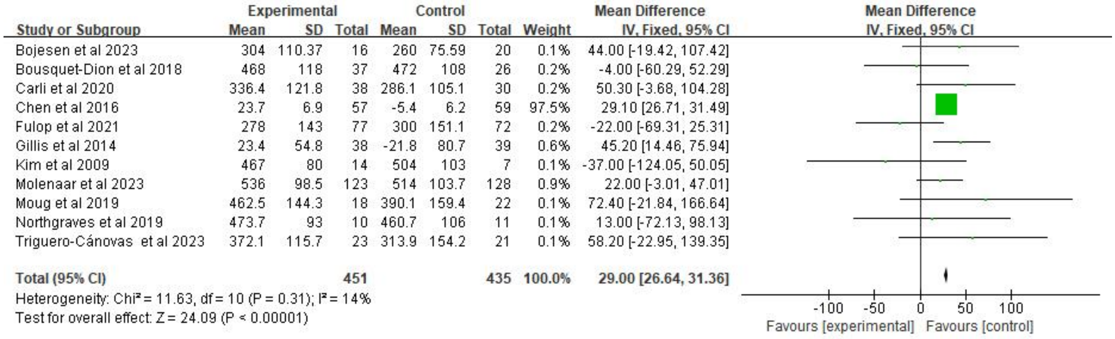

Figure S2: Subgroup analysis-6MWT

Study or Subgroup	Experimental Mean	Experimental SD	Experimental Total	Control Mean	Control SD	Control Total	Weight	Mean Difference IV, Fixed, 95% CI
**1.3.1 Multimodal prehabilitation**								
Bojesen et al 2023	304	110.37	16	260	75.59	20	0.1%	44.00 [-19.42, 107.42]
Bousquet-Dion et al 2018	468	118	37	472	108	26	0.2%	-4.00 [-60.29, 52.29]
Carli et al 2020	336.4	121.8	38	286.1	105.1	30	0.2%	50.30 [-3.68, 104.28]
Chen et al 2016	23.7	6.9	57	-5.4	6.2	59	97.5%	29.10 [26.71, 31.49]
Fulop et al 2021	278	143	77	300	151.1	72	0.2%	-22.00 [-69.31, 25.31]
Gillis et al 2014	23.4	54.8	38	-21.8	80.7	39	0.6%	45.20 [14.46, 75.94]
Molenaar et al 2023	536	98.5	123	514	103.7	128	0.9%	22.00 [-3.01, 47.01]
Triguero-Cánovas et al 2023	372.1	115.7	23	313.9	154.2	21	0.1%	58.20 [-22.95, 139.35]
**Subtotal (95% CI)**			**409**			**395**	**99.8%**	**29.03 [26.67, 31.39]**
Heterogeneity: Chi² = 8.47, df = 7 (P = 0.29); I² = 17%								
Test for overall effect: Z = 24.09 (P < 0.00001)								
**1.3.2 Exercise prehabilitation**								
Kim et al 2009	467	80	14	504	103	7	0.1%	-37.00 [-124.05, 50.05]
Moug et al 2019	462.5	144.3	18	390.1	159.4	22	0.1%	72.40 [-21.84, 166.64]
Northgraves et al 2019	473.7	93	10	460.7	106	11	0.1%	13.00 [-72.13, 98.13]
**Subtotal (95% CI)**			**42**			**40**	**0.2%**	**13.24 [-37.89, 64.36]**
Heterogeneity: Chi² = 2.79, df = 2 (P = 0.25); I² = 28%								
Test for overall effect: Z = 0.51 (P = 0.61)								
**Total (95% CI)**			**451**			**435**	**100.0%**	**29.00 [26.64, 31.36]**

Heterogeneity: Chi² = 11.63, df = 10 (P = 0.31); I² = 14%
Test for overall effect: Z = 24.09 (P < 0.00001)
Test for subgroup differences: Chi² = 0.37, df = 1 (P = 0.55), I² = 0%

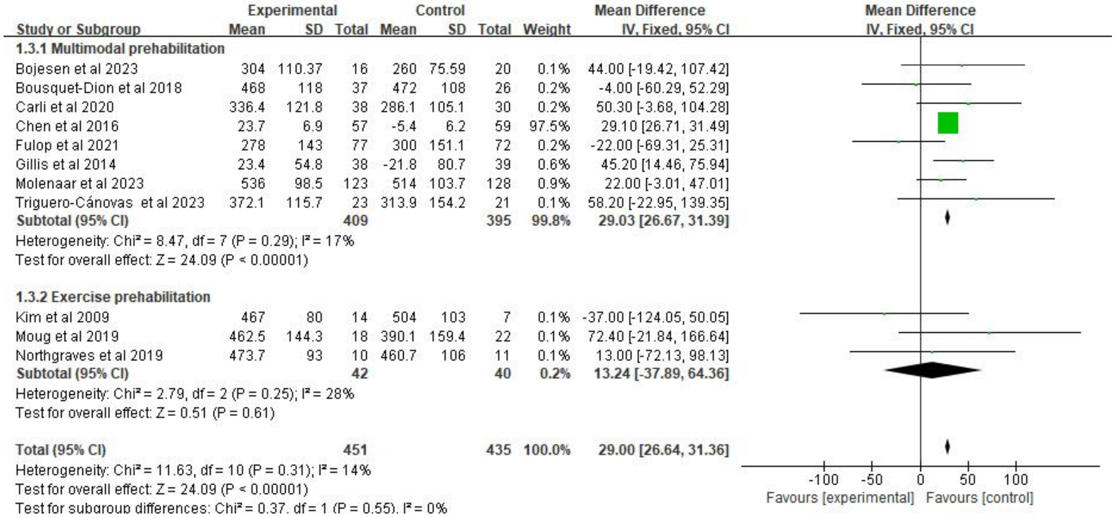

Figure S3: Hierarchical analysis of multimodal prehabilitation-6MWT

Study or Subgroup	Experimental Mean	Experimental SD	Experimental Total	Control Mean	Control SD	Control Total	Weight	Mean Difference IV, Random, 95% CI
**1.12.1 Before surgery**								
Bousquet-Dion et al 2018	470	118	37	471	108	26	4.1%	-1.00 [-57.29, 55.29]
Chen et al 2016	23.7	6.9	57	-5.4	6.2	59	22.3%	29.10 [26.71, 31.49]
Fulop et al 2021	305	143.7	77	232	110.37	72	6.6%	73.00 [32.01, 113.99]
Gillis et al 2014	25.2	50.2	38	-16.4	46	39	13.5%	41.60 [20.08, 63.12]
Kim et al 2009	467	80	14	504	103	7	1.9%	-37.00 [-124.05, 50.05]
Moug et al 2019	462.5	144.3	18	390.1	159.4	22	1.6%	72.40 [-21.84, 166.64]
**Subtotal (95% CI)**			**241**			**225**	**50.1%**	**34.77 [16.76, 52.77]**
Heterogeneity: Tau² = 190.81; Chi² = 9.79, df = 5 (P = 0.08); I² = 49%								
Test for overall effect: Z = 3.78 (P = 0.0002)								
**1.12.2 Four weeks after surgery**								
Bousquet-Dion et al 2018	441	120	37	444	116	26	3.8%	-3.00 [-62.02, 56.02]
Carli et al 2020	325.3	114.3	55	304	107.3	54	6.5%	21.30 [-20.31, 62.91]
Fulop et al 2021	256	136.3	77	276	116.3	72	6.7%	-20.00 [-60.60, 20.60]
Molenaar et al 2023	536	98.5	123	514	103.7	128	11.9%	22.00 [-3.01, 47.01]
**Subtotal (95% CI)**			**292**			**280**	**28.8%**	**10.17 [-9.82, 30.16]**
Heterogeneity: Tau² = 56.90; Chi² = 3.43, df = 3 (P = 0.33); I² = 13%								
Test for overall effect: Z = 1.00 (P = 0.32)								
**1.12.3 Six to eight weeks after surgery**								
Bousquet-Dion et al 2018	468	118	37	472	108	26	4.1%	-4.00 [-60.29, 52.29]
Fulop et al 2021	278	143	77	300	151.1	72	5.4%	-22.00 [-69.31, 25.31]
Gillis et al 2014	23.4	54.8	38	-21.8	80.7	39	9.6%	45.20 [14.46, 75.94]
Triguero-Cánovas et al 2023	372.1	115.7	23	313.9	154.2	21	2.2%	58.20 [-22.95, 139.35]
**Subtotal (95% CI)**			**175**			**158**	**21.1%**	**17.78 [-20.77, 56.33]**
Heterogeneity: Tau² = 856.73; Chi² = 7.09, df = 3 (P = 0.07); I² = 58%								
Test for overall effect: Z = 0.90 (P = 0.37)								
**Total (95% CI)**			**708**			**663**	**100.0%**	**24.17 [11.63, 36.71]**

Heterogeneity: Tau² = 181.79; Chi² = 24.37, df = 13 (P = 0.03); I² = 47%
Test for overall effect: Z = 3.78 (P = 0.0002)
Test for subgroup differences: Chi² = 3.29, df = 2 (P = 0.19), I² = 39.3%

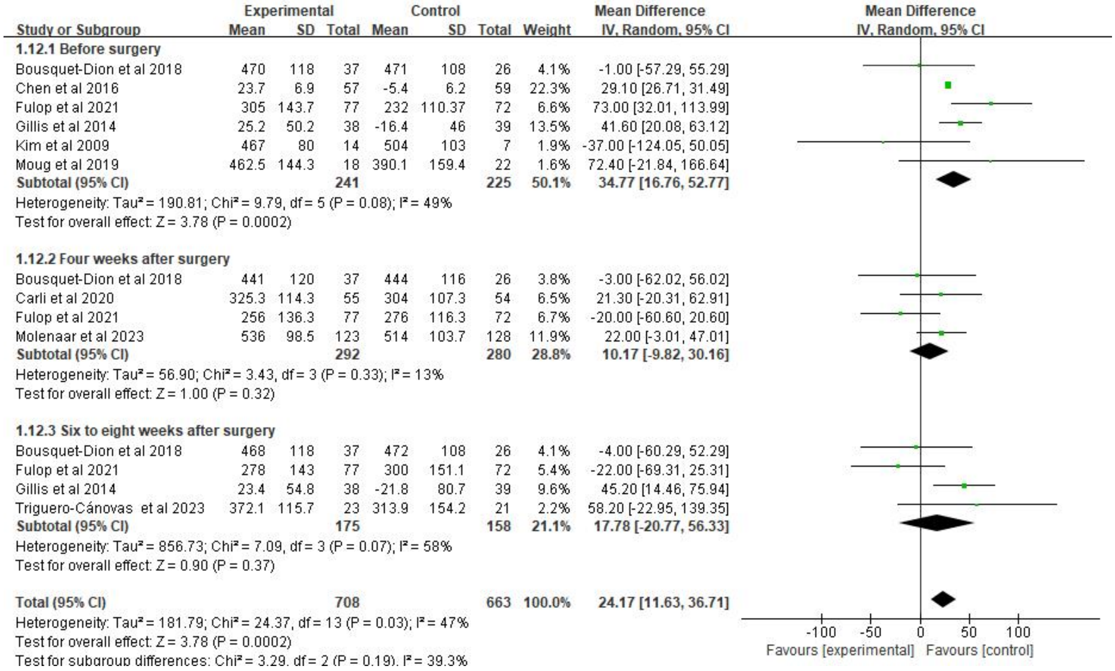

Figure S4: Overall meta-analysis- Hospital Length of stay

Study or Subgroup	Experimental Mean	Experimental SD	Experimental Total	Control Mean	Control SD	Control Total	Weight	Mean Difference IV, Fixed, 95% CI
Berkel et al 2022	8.4	7.4	28	9.1	7	29	0.2%	-0.70 [-4.44, 3.04]
Bojesen et al 2023	4	2.96	16	4	2.96	20	0.7%	0.00 [-1.95, 1.95]
Bousquet-Dion et al 2018	3	1.85	37	3	1.48	26	4.1%	0.00 [-0.82, 0.82]
Carli et al 2020	4	3.7	55	5	4.44	55	1.2%	-1.00 [-2.53, 0.53]
Fulop et al 2021	8	2.22	77	8	1.48	72	7.8%	0.00 [-0.60, 0.60]
Gillis et al 2014	4	2.22	38	5	4.44	39	1.2%	-1.00 [-2.56, 0.56]
Karlsson et al 2019	5	1.48	10	6	2.22	11	1.1%	-1.00 [-2.60, 0.60]
López-Rodríguez-Arias et al 2021	4.8	1	10	7.2	3.2	10	0.7%	-2.40 [-4.48, -0.32]
Molenaar et al 2023	3	1.48	123	3	0.74	128	33.2%	0.00 [-0.29, 0.29]
Northgraves et al 2019	8	5	10	10	7	11	0.1%	-2.00 [-7.17, 3.17]
Onerup et al 2022	9	9	317	9	8	351	1.7%	0.00 [-1.30, 1.30]
Peng et al 2021	2.43	0.87	109	2.6	0.94	104	47.4%	-0.17 [-0.41, 0.07]
Triguero-Cánovas et al 2023	5.74	3.54	23	6.67	3.49	21	0.7%	-0.93 [-3.01, 1.15]
**Total (95% CI)**			**853**			**877**	**100.0%**	**-0.14 [-0.31, 0.03]**

Heterogeneity: Chi² = 10.50, df = 12 (P = 0.57); I² = 0%
Test for overall effect: Z = 1.64 (P = 0.10)

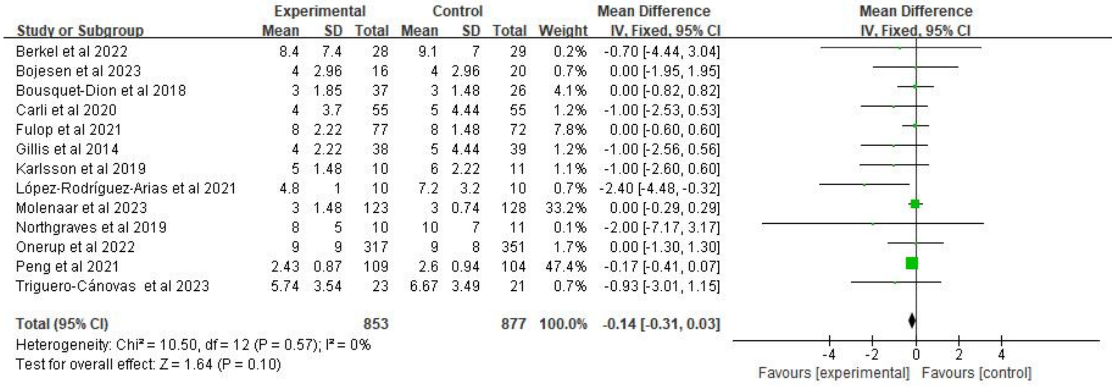

Figure S5: Subgroup analysis- Hospital Length of stay

Study or Subgroup	Experimental Mean	Experimental SD	Experimental Total	Control Mean	Control SD	Control Total	Weight	Mean Difference IV, Fixed, 95% CI
**1.10.1 Multimodal prehabilitation**								
Bojesen et al 2023	4	2.96	16	4	2.96	20	0.7%	0.00 [-1.95, 1.95]
Bousquet-Dion et al 2018	3	1.85	37	3	1.48	26	4.1%	0.00 [-0.82, 0.82]
Carli et al 2020	4	3.7	55	5	4.44	55	1.2%	-1.00 [-2.53, 0.53]
Fulop et al 2021	8	2.22	77	8	1.48	72	7.8%	0.00 [-0.60, 0.60]
Gillis et al 2014	4	2.22	38	5	4.44	39	1.2%	-1.00 [-2.56, 0.56]
Karlsson et al 2019	5	1.48	10	6	2.22	11	1.1%	-1.00 [-2.60, 0.60]
López-Rodríguez-Arias et al 2021	4.8	1	10	7.2	3.2	10	0.7%	-2.40 [-4.48, -0.32]
Molenaar et al 2023	3	1.48	123	3	0.74	128	33.2%	0.00 [-0.29, 0.29]
Onerup et al 2022	9	9	317	9	8	351	1.7%	0.00 [-1.30, 1.30]
Triguero-Cánovas et al 2023	5.74	3.54	23	6.67	3.49	21	0.7%	-0.93 [-3.01, 1.15]
**Subtotal (95% CI)**			**706**			**733**	**52.2%**	**-0.11 [-0.34, 0.12]**
Heterogeneity: Chi² = 9.79, df = 9 (P = 0.37); I² = 8%								
Test for overall effect: Z = 0.91 (P = 0.36)								
**1.10.2 Exercise prehabilitation**								
Berkel et al 2022	8.4	7.4	28	9.1	7	29	0.2%	-0.70 [-4.44, 3.04]
Northgraves et al 2019	8	5	10	10	7	11	0.1%	-2.00 [-7.17, 3.17]
Peng et al 2021	2.43	0.87	109	2.6	0.94	104	47.4%	-0.17 [-0.41, 0.07]
**Subtotal (95% CI)**			**147**			**144**	**47.8%**	**-0.18 [-0.42, 0.07]**
Heterogeneity: Chi² = 0.56, df = 2 (P = 0.76); I² = 0%								
Test for overall effect: Z = 1.42 (P = 0.15)								
**Total (95% CI)**			**853**			**877**	**100.0%**	**-0.14 [-0.31, 0.03]**

Heterogeneity: Chi² = 10.50, df = 12 (P = 0.57); I² = 0%
Test for overall effect: Z = 1.64 (P = 0.10)
Test for subgroup differences: Chi² = 0.16, df = 1 (P = 0.69), I² = 0%

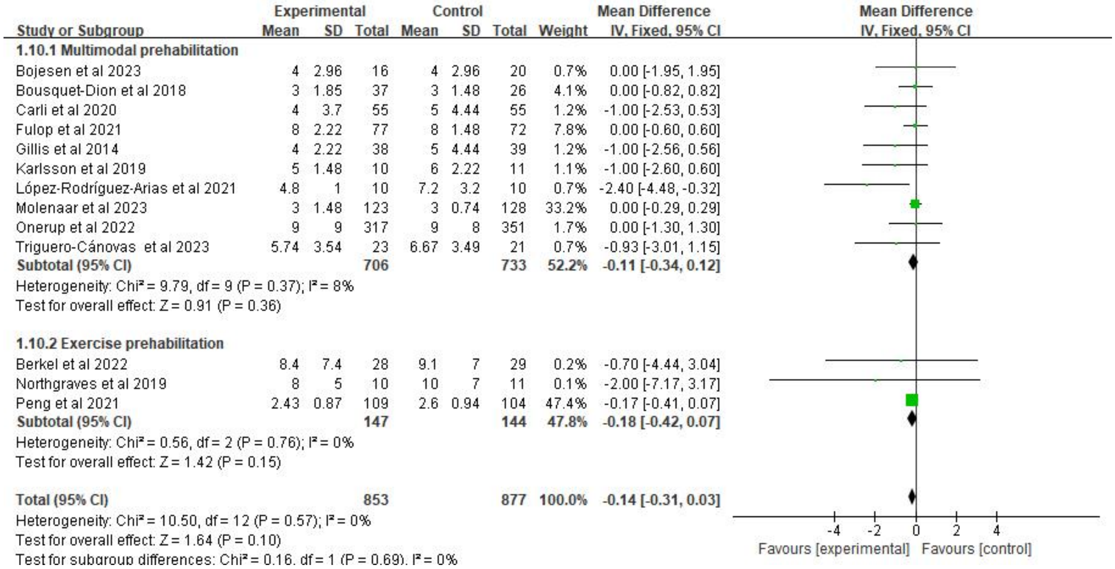

Figure S6: Overall meta-analysis- Postoperative Complications

Study or Subgroup	Experimental Events	Experimental Total	Control Events	Control Total	Weight	Risk Ratio M-H, Random, 95% CI
Berkel et al 2022	12	28	21	29	10.0%	0.59 [0.37, 0.96]
Bojesen et al 2023	10	16	19	20	12.3%	0.66 [0.44, 0.97]
Bousquet-Dion et al 2018	14	37	8	26	6.2%	1.23 [0.61, 2.50]
Carli et al 2020	25	55	25	55	11.8%	1.00 [0.66, 1.51]
Gillis et al 2014	12	38	17	39	7.9%	0.72 [0.40, 1.31]
Karlsson et al 2019	6	10	2	11	2.1%	3.30 [0.85, 12.75]
López-Rodríguez-Arias et al 2021	2	10	5	10	2.0%	0.40 [0.10, 1.60]
Molenaar et al 2023	39	123	54	128	14.1%	0.75 [0.54, 1.04]
Moug et al 2019	12	18	12	22	9.6%	1.22 [0.74, 2.02]
Onerup et al 2022	237	317	245	351	20.7%	1.07 [0.98, 1.18]
Triguero-Cánovas et al 2023	4	23	7	21	3.2%	0.52 [0.18, 1.53]
**Total (95% CI)**		**675**		**712**	**100.0%**	**0.87 [0.71, 1.07]**
Total events	373		415			

Heterogeneity: Tau² = 0.05; Chi² = 22.66, df = 10 (P = 0.01); I² = 56%
Test for overall effect: Z = 1.29 (P = 0.20)

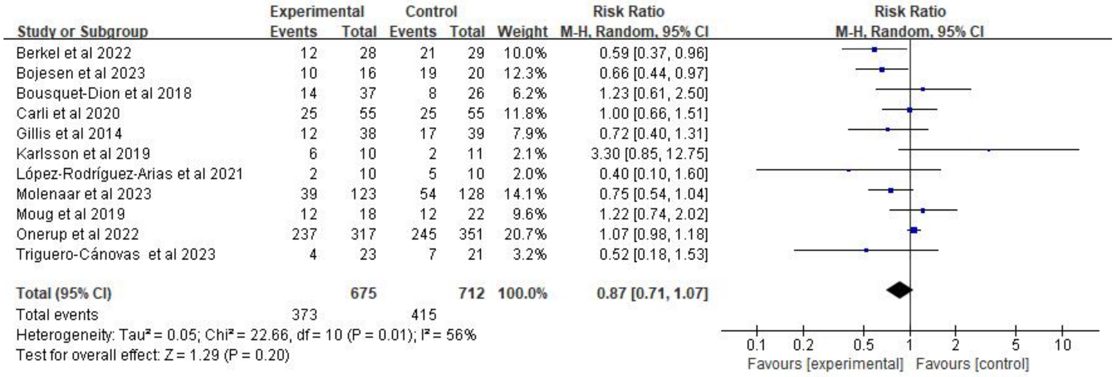

Figure S7: Subgroup analysis- Postoperative Complications

Study or Subgroup	Experimental Events	Experimental Total	Control Events	Control Total	Weight	Risk Ratio M-H, Random, 95% CI
**1.2.1 Multimodal prehabilitation**						
Bojesen et al 2023	10	16	19	20	12.3%	0.66 [0.44, 0.97]
Bousquet-Dion et al 2018	14	37	8	26	6.2%	1.23 [0.61, 2.50]
Carli et al 2020	25	55	25	55	11.8%	1.00 [0.66, 1.51]
Gillis et al 2014	12	38	17	39	7.9%	0.72 [0.40, 1.31]
López-Rodríguez-Arias et al 2021	2	10	5	10	2.0%	0.40 [0.10, 1.60]
Molenaar et al 2023	39	123	54	128	14.1%	0.75 [0.54, 1.04]
Triguero-Cánovas et al 2023	4	23	7	21	3.2%	0.52 [0.18, 1.53]
**Subtotal (95% CI)**		**302**		**299**	**57.5%**	**0.78 [0.65, 0.94]**
Total events	106		135			
Heterogeneity: Tau² = 0.00; Chi² = 5.27, df = 6 (P = 0.51); I² = 0%						
Test for overall effect: Z = 2.56 (P = 0.01)						
**1.2.2 Exercise prehabilitation**						
Berkel et al 2022	12	28	21	29	10.0%	0.59 [0.37, 0.96]
Karlsson et al 2019	6	10	2	11	2.1%	3.30 [0.85, 12.75]
Moug et al 2019	12	18	12	22	9.6%	1.22 [0.74, 2.02]
Onerup et al 2022	237	317	245	351	20.7%	1.07 [0.98, 1.18]
**Subtotal (95% CI)**		**373**		**413**	**42.5%**	**1.03 [0.70, 1.51]**
Total events	267		280			
Heterogeneity: Tau² = 0.09; Chi² = 8.66, df = 3 (P = 0.03); I² = 65%						
Test for overall effect: Z = 0.13 (P = 0.90)						
**Total (95% CI)**		**675**		**712**	**100.0%**	**0.87 [0.71, 1.07]**
Total events	373		415			
Heterogeneity: Tau² = 0.05; Chi² = 22.66, df = 10 (P = 0.01); I² = 56%						
Test for overall effect: Z = 1.29 (P = 0.20)						
Test for subgroup differences: Chi² = 1.56, df = 1 (P = 0.21), I² = 35.7%						

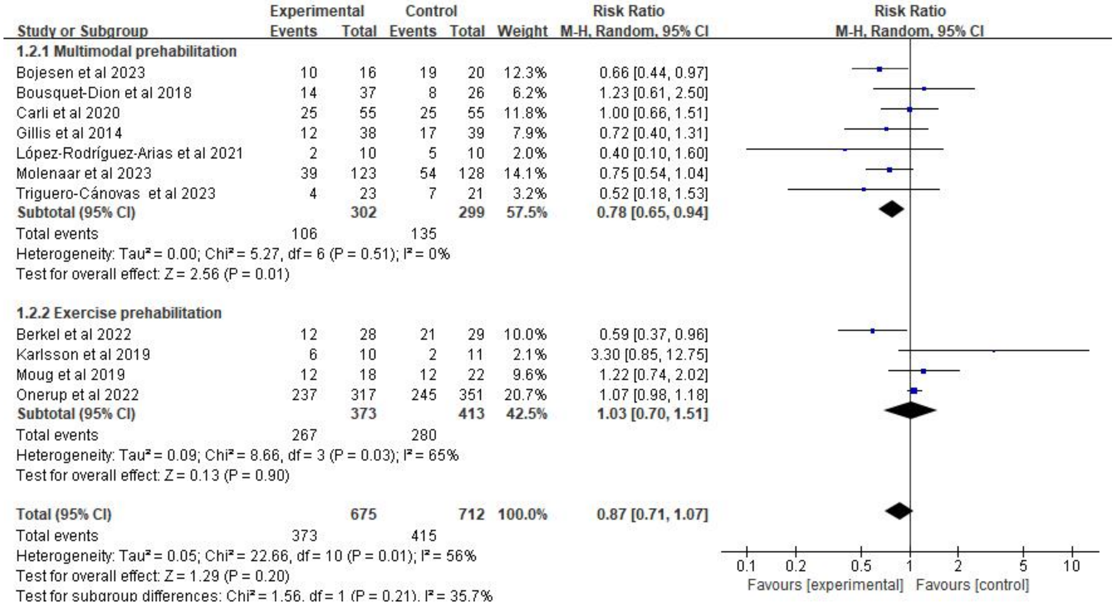

Figure S8: Overall meta-analysis-Anxiety scores

Study or Subgroup	Experimental Mean	Experimental SD	Experimental Total	Control Mean	Control SD	Control Total	Weight	Mean Difference IV, Fixed, 95% CI
Carli et al 2020	4	3.7	37	4	5.19	31	10.4%	0.00 [-2.18, 2.18]
Fulop et al 2021	4	3	77	5	3	72	53.1%	-1.00 [-1.96, -0.04]
Gillis et al 2014	4.3	3.4	38	5.1	4.7	39	14.7%	-0.80 [-2.63, 1.03]
López-Rodríguez-Arias et al 2021	5.3	4.36	20	5.3	4.36	20	6.8%	0.00 [-2.70, 2.70]
Taha et al 2021	2.8	3.1	23	3.2	3.3	25	15.0%	-0.40 [-2.21, 1.41]
**Total (95% CI)**			**195**			**187**	**100.0%**	**-0.71 [-1.41, -0.01]**
Heterogeneity: Chi² = 1.14, df = 4 (P = 0.89); I² = 0%								
Test for overall effect: Z = 1.98 (P = 0.05)								

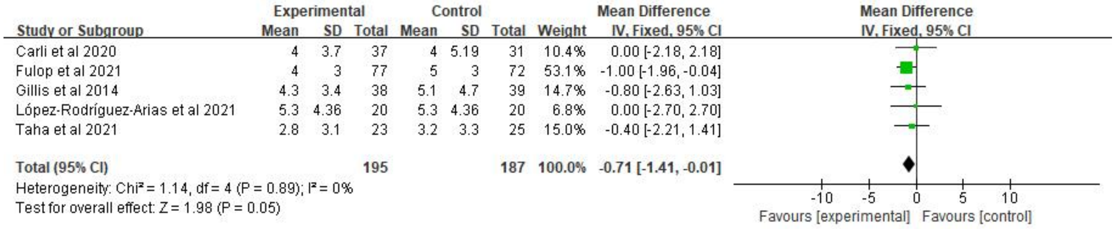

Figure S9: Subgroup analysis- Anxiety scores

Study or Subgroup	Experimental Mean	Experimental SD	Experimental Total	Control Mean	Control SD	Control Total	Weight	Mean Difference IV, Fixed, 95% CI
**1.5.1 Multimodal prehabilitation**								
Carli et al 2020	4	3.7	37	4	5.19	31	10.4%	0.00 [-2.18, 2.18]
Fulop et al 2021	4	3	77	5	3	72	53.1%	-1.00 [-1.96, -0.04]
Gillis et al 2014	4.3	3.4	38	5.1	4.7	39	14.7%	-0.80 [-2.63, 1.03]
López-Rodríguez-Arias et al 2021	5.3	4.36	20	5.3	4.36	20	6.8%	0.00 [-2.70, 2.70]
**Subtotal (95% CI)**			**172**			**162**	**85.0%**	**-0.76 [-1.53, -0.00]**
Heterogeneity: Chi² = 1.01, df = 3 (P = 0.80); I² = 0%								
Test for overall effect: Z = 1.96 (P = 0.05)								
**1.5.2 Exercise prehabilitation**								
Taha et al 2021	2.8	3.1	23	3.2	3.3	25	15.0%	-0.40 [-2.21, 1.41]
**Subtotal (95% CI)**			**23**			**25**	**15.0%**	**-0.40 [-2.21, 1.41]**
Heterogeneity: Not applicable								
Test for overall effect: Z = 0.43 (P = 0.67)								
**Total (95% CI)**			**195**			**187**	**100.0%**	**-0.71 [-1.41, -0.01]**
Heterogeneity: Chi² = 1.14, df = 4 (P = 0.89); I² = 0%								
Test for overall effect: Z = 1.98 (P = 0.05)								
Test for subgroup differences: Chi² = 0.13, df = 1 (P = 0.72), I² = 0%								

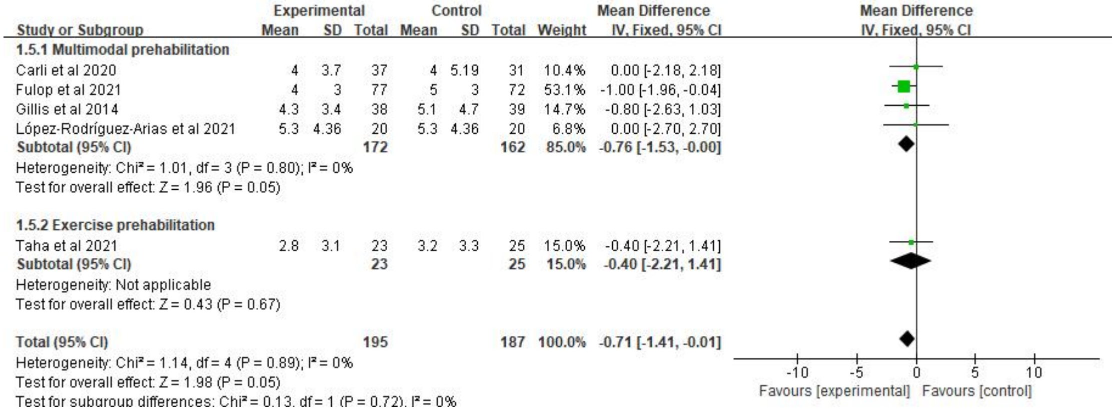

Figure S10: Overall meta-analysis- depression scores

Study or Subgroup	Experimental Mean	Experimental SD	Experimental Total	Control Mean	Control SD	Control Total	Weight	Std. Mean Difference IV, Fixed, 95% CI
Carli et al 2020	4	2.22	37	4	4.44	31	16.2%	0.00 [-0.48, 0.48]
Fulop et al 2021	4	4	77	5	4	72	35.6%	-0.25 [-0.57, 0.07]
Gillis et al 2014	3.2	3.2	38	3.7	4.3	39	18.5%	-0.13 [-0.58, 0.32]
López-Rodríguez-Arias et al 2021	4	4	20	4	4	20	9.6%	0.00 [-0.62, 0.62]
Moug et al 2019	8.1	6.9	15	7	5.2	22	8.6%	0.18 [-0.48, 0.84]
Taha et al 2021	1.6	1.9	23	1.8	3.7	25	11.5%	-0.07 [-0.63, 0.50]
**Total (95% CI)**			**210**			**209**	**100.0%**	**-0.10 [-0.30, 0.09]**
Heterogeneity: Chi² = 1.82, df = 5 (P = 0.87); I² = 0%								
Test for overall effect: Z = 1.07 (P = 0.29)								

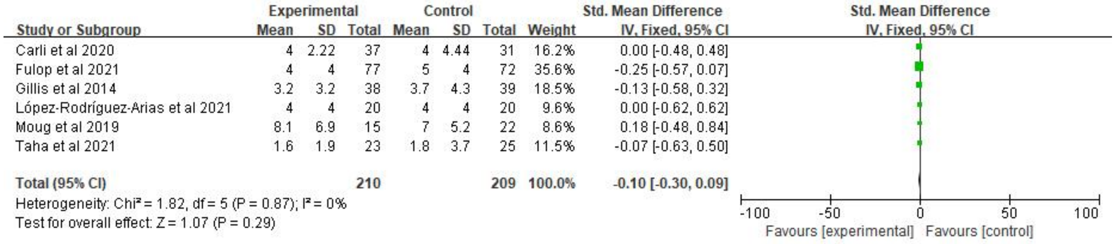

Figure S11: Subgroup analysis- depression scores

Study or Subgroup	Experimental Mean	Experimental SD	Experimental Total	Control Mean	Control SD	Control Total	Weight	Std. Mean Difference IV, Fixed, 95% CI
**1.6.1 Multimodal prehabilitation**								
Carli et al 2020	4	2.22	37	4	4.44	31	16.2%	0.00 [-0.48, 0.48]
Fulop et al 2021	4	4	77	5	4	72	35.6%	-0.25 [-0.57, 0.07]
Gillis et al 2014	3.2	3.2	38	3.7	4.3	39	18.5%	-0.13 [-0.58, 0.32]
López-Rodríguez-Arias et al 2021	4	4	20	4	4	20	9.6%	0.00 [-0.62, 0.62]
**Subtotal (95% CI)**			**172**			**162**	**79.9%**	**-0.14 [-0.36, 0.07]**
Heterogeneity: Chi² = 0.96, df = 3 (P = 0.81); I² = 0%								
Test for overall effect: Z = 1.28 (P = 0.20)								
**1.6.2 Exercise prehabilitation**								
Moug et al 2019	8.1	6.9	15	7	5.2	22	8.6%	0.18 [-0.48, 0.84]
Taha et al 2021	1.6	1.9	23	1.8	3.7	25	11.5%	-0.07 [-0.63, 0.50]
**Subtotal (95% CI)**			**38**			**47**	**20.1%**	**0.04 [-0.39, 0.47]**
Heterogeneity: Chi² = 0.31, df = 1 (P = 0.58); I² = 0%								
Test for overall effect: Z = 0.18 (P = 0.86)								
**Total (95% CI)**			**210**			**209**	**100.0%**	**-0.10 [-0.30, 0.09]**
Heterogeneity: Chi² = 1.82, df = 5 (P = 0.87); I² = 0%								
Test for overall effect: Z = 1.07 (P = 0.29)								
Test for subgroup differences: Chi² = 0.54, df = 1 (P = 0.46), I² = 0%								

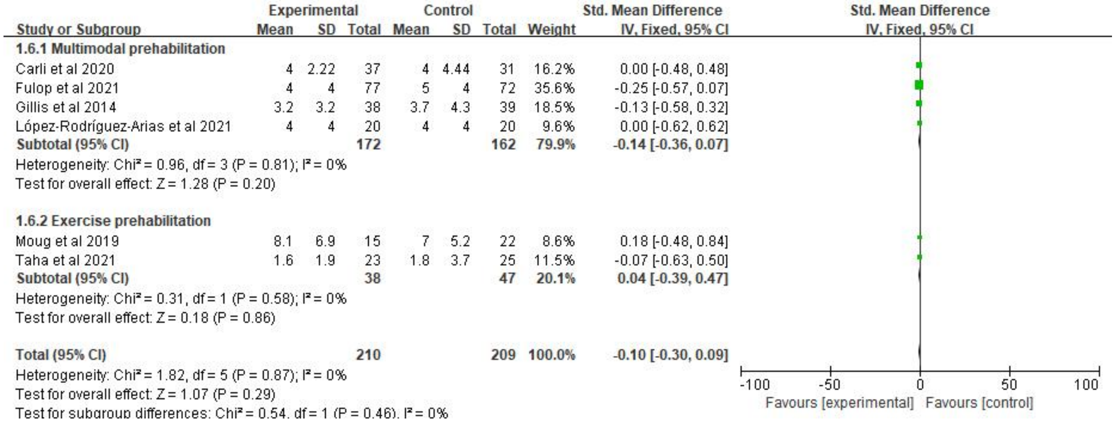

Figure S12: Publication bias

6MWT

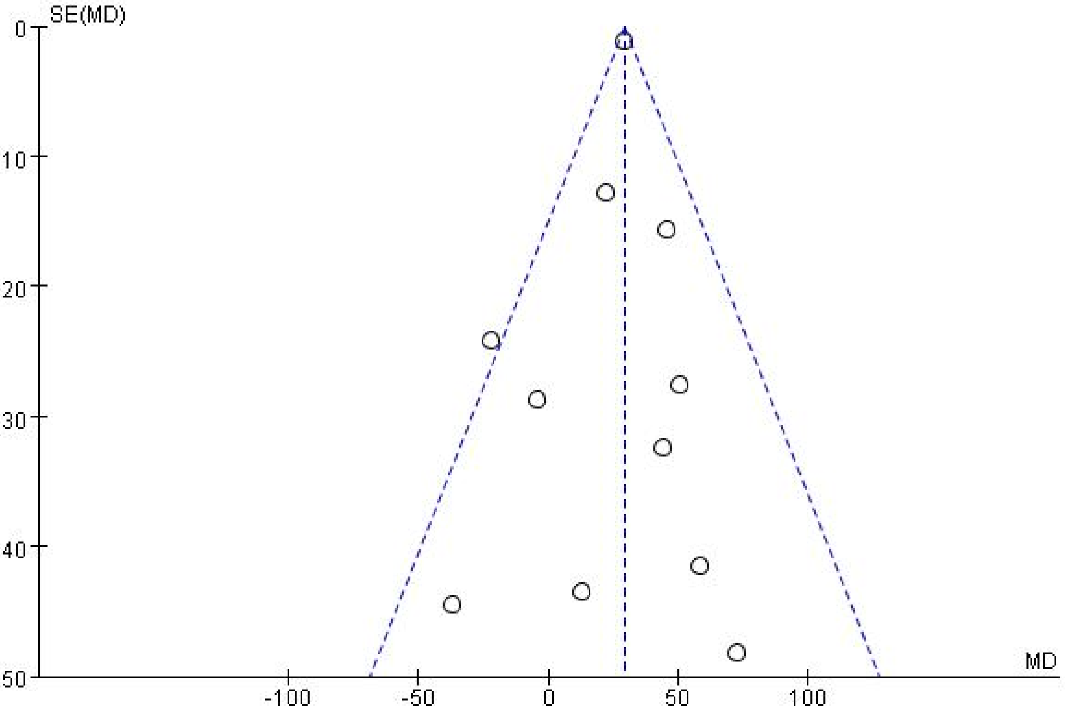

Hospital Length of Stay

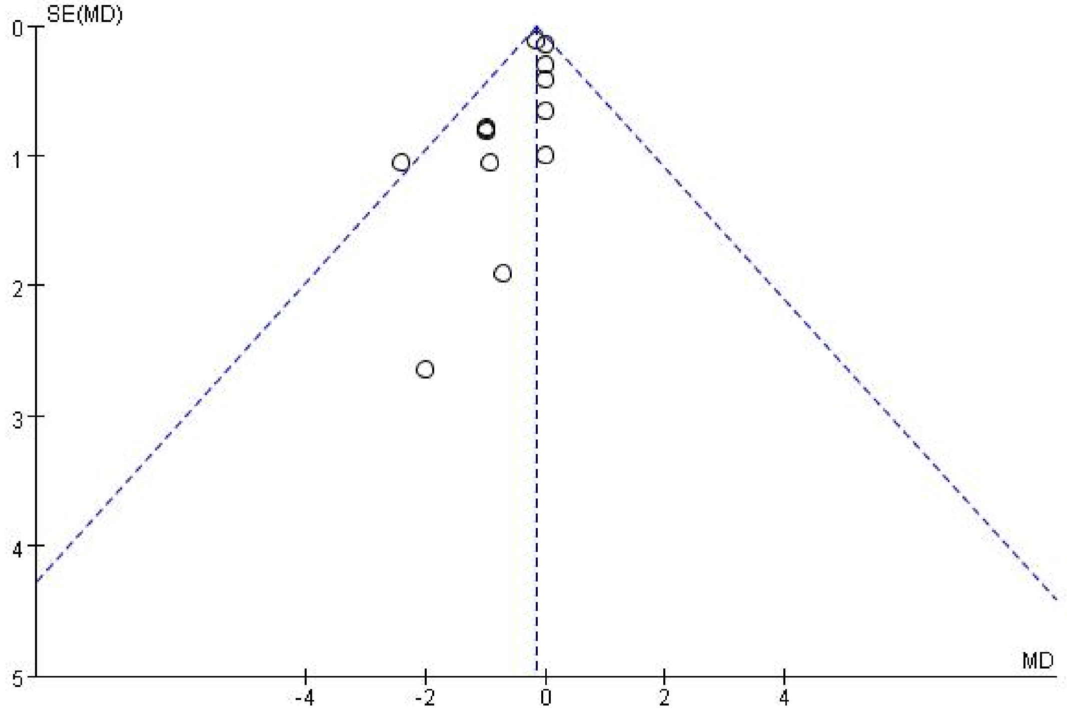

Postoperative Complications

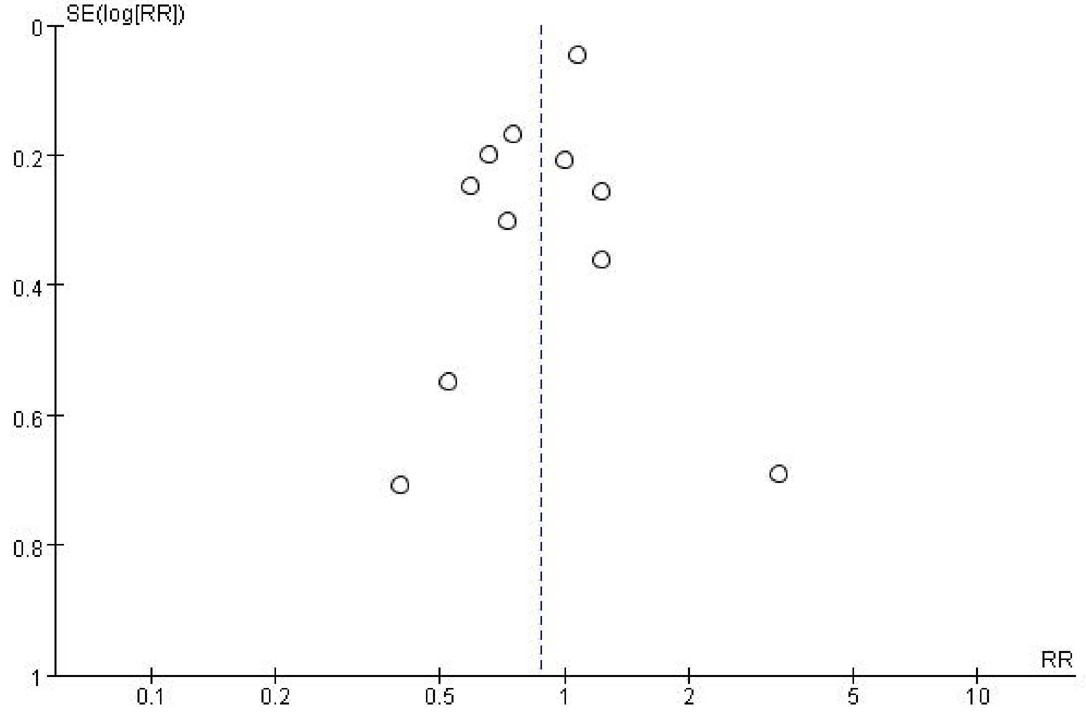

Table S1: Risk of bias assessment

Author, year	Domain 1	Domain 2	Domain 3	Domain 4	Domain 5	Overall ROB
Carli et al 2020	Low	Low	Low	Low	Low	Low
Molenaar et al 2023	Low	Low	Low	Low	Low	Low
Fulop et al 2021	SC	Low	Low	Low	Low	SC
Berkel et al 2022	Low	Low	Low	Low	Low	Low
Gillis et al 2014	Low	Low	Low	Low	Low	Low
Peng et al 2021	Low	Low	Low	Low	Low	Low
Moug et al 2019	Low	Low	Low	Low	Low	Low
Bousquet-Dion et al 2018	SC	Low	Low	Low	Low	SC
López-Rodríguez-Arias et al 2021	SC	Low	Low	Low	Low	SC
Northgraves et al 2020	Low	Low	Low	Low	Low	Low
Taha et al 2021	Low	Low	Low	Low	Low	Low
Chen et al 2017	SC	Low	Low	Low	Low	SC
Kim et al 2009	SC	Low	Low	Low	SC	SC
Onerup et al 2022	SC	Low	Low	Low	Low	SC
Karlsson et al 2019	SC	Low	Low	Low	Low	SC
Triguero-Cánovas et al 2023	SC	Low	Low	Low	Low	SC
Bojesen et al 2023	SC	Low	Low	Low	Low	SC

Domain 1: bias arising from the randomization process, Domain 2: bias due to deviations from intended interventions, Domain 3: bias due to missing outcome data, Domain 4: bias in measurement of the outcome, Domain 5: bias in selection of the reported result, L: low risk of bias, H: high risk of bias, SC: some concern.

Table S2: Summary of the results of sensitivity analyses

Sensitivity analyses- risk bias of the studies			
Outcomes	Subgroups	Removed study	Statistical results
6MWT	Exercise prehabilitation	Kim et al 2009	(MD = 39.69, 95 % CI [-23.48, 102.86], P =0.22)
	Multimodal prehabilitation	Bousquet-Dion et al 2018, Chen et al 2017, Fulop et al 2021, Triguero-Cánovas et al 2023, Bojesen et al 2023	(MD = 39.42, 95 % CI [15.16, 51.68], P < 0.001)
Hospital length of stay	Exercise prehabilitation	Not applicable	(MD = -0.18, 95 % CI [-0.42, 0.07], P =0.15)
	Multimodal prehabilitation	Bousquet-Dion et al 2018, Fulop et al 2021, Karlsson et al 2019, Onerup et al 2022, López-Rodríguez-Arias et al 2021, Triguero-Cánovas et al 2023, Bojesen et al 2023	(MD = -0.07, 95 % CI [-0.35, 0.22], P =0.64)
Postoperative Complications	Exercise prehabilitation	Karlsson et al 2019, Onerup et al 2022	(RR = 0.85, 95 % CI [0.41, 1.73], P =0.65)
	Multimodal prehabilitation	Bousquet-Dion et al 2018, López-Rodríguez-Arias et al 2021, Triguero-Cánovas et al 2023, Bojesen et al 2023	(RR = 0.82, 95 % CI [0.65 1.04], P =0.10)
Anxiety scores	Exercise prehabilitation	Not applicable	(MD = -0.40, 95 % CI [-2.21, 1.41], P =0.05)
	Multimodal prehabilitation	Fulop et al 2021, López-Rodríguez-Arias et al 2021	(MD = -0.47, 95 % CI [-1.87, 0.93], P =0.51)
Depression scores	Exercise prehabilitation	Not applicable	(MD = 0.04, 95 % CI [-0.39, 0.47], P =0.86)
	Multimodal prehabilitation	Fulop et al 2021, López-Rodríguez-Arias et al 2021	(SMD = -0.07, 95 % CI [-0.40., 0.26], P =0.68)
Sensitivity analyses-population characteristics			
Outcomes	Subgroups	Removed study	Statistical results
6MVT	Exercise prehabilitation	Moug et al 2019	(MD = -11.44, 95 % CI[-72.30, 49.42] , P =0.710)
	Multimodal prehabilitation	Carli et al 2020, Chen et al 2016	(MD = 24.00, 95 % CI 24.00 [7.82, 40.19], P =0.004)
Hospital length of stay	Exercise prehabilitation	Berkel et al 2022	(MD = -0.17, 95 % CI[-0.42, 0.07] , P =0.16)
	Multimodal prehabilitation	Carli et al 2020	(MD = -0, 09, 95 % CI [-0.32, 0.15], P =0.47)
Postoperative Complications	Exercise prehabilitation	Berkel et al 2022, Moug et al 2019	(MD = 1.53, 95 % CI [0.54, 4.32], P =0.42)
	Multimodal prehabilitation	Carli et al 2020	(MD = 0.73, 95 % CI [0.59, 0.90], P =0.04)

Anxiety scores	Exercise prehabilitation	Not applicable	Not applicable
	Multimodal prehabilitation	Carli et al 2020	(SMD = -0.87, 95 % CI [-1.68, -0.06], P =0.04)
Depression scores	Exercise prehabilitation	Moug et al 2019	(SMD = -0.07, 95 % CI [-0.63, 0.50], P =0.82)
	Multimodal prehabilitation	Carli et al 2020	(SMD = -0.18, 95 % CI [-0.42, 0.06], P =0.15)

RR-risk ratio; MD- mean difference; SMD- standardized mean difference; CI- confidence interval; 6MWT-the 6-minute walk test

Table S3: GRADE evidence profile

Outcomes	Subgroups	Types of original studies	RR (95% CI)	Mean Difference 95%CI	Limitations in study design	Inconsistency	Imprecision	Indirectness	Publication bias	Quality of evidence
6MVT	Exercise prehabilitation	RCTs (3)	***	13.24 [-37.89, 64.36]	---	↓	↓	---	↓	Very low
	Multimodal prehabilitation	RCTs (8)	***	29.03 [26.67, 31.39]	---	↓	---	---	↓	Low
Hospital length of stay	Exercise prehabilitation	RCTs (3)	***	-0.18 [-0.42, 0.07]	---	---	---	---	↓	Moderate
	Multimodal prehabilitation	RCTs (10)	***	-0.10 [-0.34, 0.12]	---	---	---	---	↓	Moderate
Postoperative complications	Exercise prehabilitation	RCTs (4)	1.03 [0.70, 1.51]	***	---	↓	---	---	↓	Low
	Multimodal prehabilitation	RCTs (7)	0.78 [0.65, 0.94]	***	---	---	---	---	↓	Moderate
Anxiety scores	Exercise prehabilitation	RCTs (1)	***	-0.40 [-2.21, 1.41]			Not applicable			
	Multimodal prehabilitation	RCTs (4)	***	-0.76 [-1.53, -0.00]	---	---	↓	---	↓	Low
Depression scores	Exercise prehabilitation	RCTs (2)	***	0.04 [-0.39, 0.47]	---	↓	---	--	↓	Low
	Multimodal prehabilitation	RCTs (4)	***	-0.14 [-0.36, 0.07]	---	---	↓	---	↓	Low

GRADE- grading of Recommendations, Assessment, Development and Evaluation; RCTs-randomized controlled trials
